# A Comparison of Screening Methods to Identify Waterlogging Tolerance in the Field in *Brassica napus* L. during Plant Ontogeny

**DOI:** 10.1371/journal.pone.0089731

**Published:** 2014-03-03

**Authors:** Xiling Zou, Chengwei Hu, Liu Zeng, Yong Cheng, Mingyue Xu, Xuekun Zhang

**Affiliations:** Key Laboratory of Oil Crop Biology and Genetic Improvement of the Ministry of Agriculture, Oil Crops Research Institute of the Chinese Academy of Agricultural Sciences, Wuhan, Hubei, China; Key Laboratory of Horticultural Plant Biology (MOE), China

## Abstract

Waterlogging tolerance is typically evaluated at a specific development stage, with an implicit assumption that differences in waterlogging tolerance expressed in these systems will result in improved yield performance in fields. It is necessary to examine these criteria in fields.

In the present study, three experiments were conducted to screen waterlogging tolerance in 25 rapeseed (*Brassica napus* L.) varieties at different developmental stages, such as seedling establishment stage and seedling stage at controlled environment, and maturity stage in the fields. The assessments for physiological parameters at three growth stages suggest that there were difference of waterlogging tolerance at all the development stages, providing an important basis for further development of breeding more tolerant materials. The results indicated that flash waterlogging restricts plant growth and growth is still restored after removal of the stress. Correlation analysis between waterlogging tolerance coefficient (WTC) of yield and other traits revealed that there was consistency in waterlogging tolerance of the genotypes until maturity, and good tolerance at seedling establishment stage and seedling stage can guarantee tolerance in later stages. The waterlogging-tolerant plants could be selected using some specific traits at any stage, and selections would be more effective at the seedling establishment stage.

Thus, our study provides a method for screening waterlogging tolerance, which would enable the suitable basis for initial selection of a large number of germplasm or breeding populations for waterlogging tolerance and help for verifying their potential utility in crop-improvement.

## Introduction

Waterlogging is an important abiotic determinant for crop growth [1]. A major feature of waterlogging is depletion of oxygen [2], which results in changes in the metabolism and morphology of plants. The formation of aerenchyma is an important adaptive response of waterlogged plants [3], [4]. During waterlogging, plant growth is either inhibited or enhanced based on the types of species and survival strategy [5]. Photosynthesis is also inhibited due to stomatal closure, leaf chlorophyll degradation or early leaf senescence [6], [7]. Moreover, waterlogging usually causes major or even total yield loss for crops [8], [9].

Yield loss under waterlogging can be reduced by controlling the drainage in fields. However, this process is labor, time and cost intensive, which makes the waterlogging stress inevitable. To study the genetic mechanism underlying natural variation in waterlogging tolerance of species is an important step towards the waterlogging management. Before looking for an answer to the question “Why are tolerant genotypes tolerant”, the key issue is to develop a selection criterion to answer the question “Which genotypes are tolerant?”.

Previous studies have outlined methods that depend on traits that help in screening plants for waterlogging tolerance. When natural variation of tolerance among Arabidopsis accessions was screened, the median lethal times of 10-leaf seedlings subjected to complete submergence in the dark was estimated as a measure of tolerance [10]. Another study about phenotypic plasticity in response to flooded conditions in Arabidopsis measured three sets of traits, including vegetative, architectural and reproductive [11]. Leaf color, plant height, and growth rate were evaluated during the screening of 99 accessions of perennial ryegrass under submergence and recovery conditions [12]. Waterlogging injury scores were used for the identification of QTLs underlying waterlogging tolerance in soybean [13]. Qiu performed QTL mapping associated with waterlogging tolerance during seedling stage in maize using traits of root length, root dry weight, plant height, shoot dry weight, and total dry weight [14], and similar work were carried out in wheat [8]. In rice, Sub1A is mapped based cloned from rice by evaluating leaf death on individual plant after one week of recovery followed completely submerged [15], and SNORKEL1 and SNORKEL2 were cloned by using the trait of early elongation ability adapting to deep water [16]. Most of these studies were performed with seedlings growing in green house during treatments, and the traits of seedling were evaluated, with the implicit assumption that differences in waterlogging tolerance expressed in these systems would result in improved yield performance in the field. However, it is necessary to compare screening methods and criterions under controlled conditions to yield loss in the fields under waterlogging.

Rapeseed is the second largest oil crop in the world. In China, the largest planting country in the world, 80% of rapeseed is planted along the Yangtze River. In this area, rapeseed is planted in paddy fields as a rotation crop just after rice [17]. Owing to the lack of labor required for transplanting, direct seeding of rapeseed is increasingly being practiced by farmers, which can result in severe waterlogging. Heavy rainfall during this season further compounds the problem. Waterlogging always results in yield loss in rapeseed. However, there have been only a handful of studies on screening of waterlogging tolerance in rapeseed.

Therefore, the present study was conducted to compare the efficacy of various methods at different growth stages under artificial environments and in fields based on traits associated with waterlogging tolerance. We address the following questions: (1) is there genetic variation for yield loss among genotypes after being exposed to waterlogging in fields? (2) Is there plasticity and variation for waterlogging tolerance-related traits at the seedling establishment and seedling stage under controlled conditions? (3) Is it possible to screen at seedling establishment and seedling stages to identify differences in waterlogging tolerance in the fields?

## Materials and Methods

Twenty-five varieties were selected for this study, including ZY821, YG2009, HYZ9, ZS9, ZS7, ZS12, Y108, 9548, 9508, 9981, B108, 21937, H0102, 95_3_12, Y208, JHTG, JHY201, GH01, HYZ62, HYZ6, FS9, HS3, NY1, and QY8 (supplied by Oil Crops Research Institute of the Chinese Academy of Agricultural Sciences).

### Experiment 1: screening at maturity stage in the field

The field trials were conducted in two different environments: Xishui, and Wuxue, Hubei in China, which is managed by local people. The owners provided full access permissions to the study sites. This study did not involve specimen collection. The field studies did not involve any threatened or endangered species. No vertebrates were involved in this study.

Planting was conducted using a randomized complete-block design with two replicates in each environment. Seeds were directly sown in the fields at the end of September. Each plot was 13.2 m^2^ (6.6 m×2 m). Plants were thinned to 450 individuals for one replicate and the plant density was about 33–37 plants m^−2^. The field management followed standard agricultural practice. Waterlogging treatment was imposed at the seedling stage with 4–6 leaves. All the fields of treatment plots were filled with water to 1 cm above the soil surface, which was maintained for 15 d.

The plants were harvested at the beginning of the following May. In each replicate, 10 representative individuals from the middle of each plot were harvested at physiological maturity. The traits evaluated included plant height (*Ph*), branch height (*Br*), primary branches (*Pbr*), main shoot length (*Msl*), siliques on the main shoot (*Sqms*), siliques on branches (*Sqb*), siliques on a plant (*Sqp*), seeds in a siliques (*Ssq*) and root length. Primary and secondary branches were the number of branches arising from the main shoot and primary branches, respectively. Main shoot length was measured from the base of the last primary branch to the tip of the main shoot. Siliques on the main shoot and siliques on a plant were the number of well-filled, normal siliques in the main shoot and the whole plant, respectively. Seeds in a silique were counted as the number of well-developed seeds in a silique. Phenotyping was done from the ten selected plants. Moreover, the number of all the plants in each plot (*Sn*, seedlings number) and the yield in each plot were also evaluated.

Waterlogging tolerance coefficient (WTC) was used to measure waterlogging tolerance. WTC of samples was calculated using the following formula:
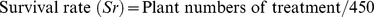


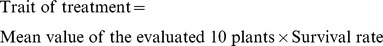


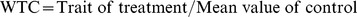



### Experiment 2: screening at the seedling establishment stage

Healthy seeds of each variety were selected and were placed on moist filter paper. Seeds were grown at 25°C (light∶dark, 16∶8 h) until their radicals were about 2–5 mm. Uniform seeds were selected and divided into two groups. One group was cultured with a normal water supply as the control and the other was followed by treatment. Thirty normal germinated seeds were treated for 12 h in a sealed tube completely filled with distilled water to avoid oxygen exchange with the air. Following removal from anaerobic chambers, seeds were sown in dishes filled with moist fine vermiculite and were cultured for 5 d.

All the seedlings of each variety per replicate were used for measuring of root length (*Rl*), shoot length (*Sl*), full length (*Fl*) and full weight (*Fw*) under control and waterlogged conditions.

WTC of samples was calculated using the following formula:
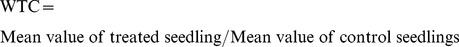



### Experiment 3: screening at the seedling stage in the green house

Uniform-sized seeds of each variety were sown in pots and the seedlings were thinned to ten per pot after 5 d. The experiment was conducted under the same growth conditions as described in experiment 2.

Each variety included control and waterlogging treatment. The experimental design was a split-plot design with three replications. All pots of the treatment were filled with water to 2–3 cm above the soil surface at the seedling stage with three leaves, which was maintained for 5 d. After waterlogging, the treated seedlings of treatment were returned to normal conditions to continue growing. Three days later, the data for traits from both treatment and control were recorded from these seedlings, including root length (*Rl*), shoot length (*Sl*), full length (*Fl*), root weight (*Rw*), shoot weight (*Sw*), weight and full weight (*Fw*).

WTC of samples was calculated using the following formula:
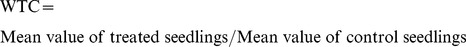



### Statistical analysis

The means of all observations were calculated using Microsoft Excel. The relative analysis and the linear relativity were performed using SAS software [18].

## Results

### The effect on yield related traits by waterlogging

To study the effect of waterlogging on yield, the seedlings were waterlogged in fields and yield related traits were compared to those of control. Under normal condition, all the evaluated traits except of branch height, main shoot length and root length were significantly related with yield (Table 1), while all the evaluated traits were significantly related with yield under waterlogging condition (Table 2). Besides, there was significantly correlation between WTC of any two traits under waterlogging (Table 3).

**Table 1:** Correlation coefficients (*r*) between pairs of yield related traits under control.

	Ph	Bh	Msl	Pb	Tb	Sqms	Sqb	Sqp	Ssq	Rl	Yield
**Ph**	1	0.701***	0.212	0.530**	0.481*	0.471*	0.562**	0.5750**	−0.159	−0.158	0.486**
**Bh**		1	−0.173	0.157	−0.010	0.077	0.080	0.137	0.101	0.066	0.326
**Msl**			1	−0.159	0.011	0.298	0.049	0.042	−0.243	0.077	−0.159
**Pb**				1	0.915***	0.437*	0.868***	0.761***	−0.003	−0.358	0.382
**Tb**					1	0.4960*	0.891***	0.681***	−0.221	−0.388	0.315
**Sqms**						1	0.707***	0.729***	−0.223	−0.224	0.552**
**Sqb**							1	0.914***	−0.088	−0.367	0.485*
**Sqp**								1	0.042	−0.283	0.557**
**Ssq**									1	0.005	0.145
**Rl**										1	−0.209
**Yield**											1

**Table 2:** Correlation coefficients (*r*) between pairs of yield related traits after waterlogging.

	Ph	Bh	Msl	Pb	Tb	Sqms	Sqb	Sqp	Ssq	Rl	Yield	Sn
**Ph**	1	0.943***	0.932***	0.500***	0.897***	0.986***	0.760***	0.922***	0.940***	0.938***	0.853***	0.879***
**Bh**		1	0.939***	0.934***	0.882***	0.938***	0.785***	0.913***	0.795***	0.849***	0.804***	0.823***
**Msl**			1	0.853***	0.795***	0.926***	0.690***	0.852***	0.806***	0.879***	0.767***	0.796***
**Pb**				1	0.974***	0.919***	0.903***	0.971***	0.826***	0.826***	0.817***	0.819***
**Tb**					1	0.871***	0.879***	0.934***	0.807***	0.801***	0.798***	0.781***
**Sqms**						1	0.757***	0.927***	0.937***	0.936***	0.867***	0.868***
**Sqb**							1	0.946***	0.615**	0.598***	0.739***	0.610***
**Sqp**								1	0.814***	0.802***	0.854***	0.778***
**Ssq**									1	0.953***	0.828***	0.813***
**Rl**										1	0.772***	0.816***
**Yield**											1	0.600**
**Sn**												1

**Table 3:** Correlation coefficients (*r*) between pairs of WTC of yield related traits.

	Ph	Bh	Msl	Pb	Tb	Sqms	Sqb	Sqp	Ssq	Rl	Sr	Yield
**Ph**	1	0.926***	0.946***	0.904***	0.858***	0.982***	0.777***	0.897***	0.924***	0.960***	0.871***	0.871***
**Bh**		1	0.949***	0.846***	0.772***	0.902***	0.715***	0.819***	0.754***	0.860***	0.811***	0.729***
**Msl**			1	0.810***	0.744***	0.901***	0.704***	0.815***	0.815***	0.911***	0.793***	0.773***
**Pb**				1	0.970***	0.930***	0.908***	0.947***	0.819***	0.845***	0.792***	0.855***
**Tb**					1	0.878***	0.849***	0.895***	0.836***	0.821***	0.748***	0.850***
**Sqms**						1	0.834***	0.940***	0.905***	0.934***	0.843***	0.893***
**Sqb**							1	0.962***	0.657***	0.678***	0.580**	0.859***
**Sqp**								1	0.800***	0.820***	0.706***	0.933***
**Ssq**									1	0.929***	0.833***	0.868***
**Rl**										1	0.840***	0.818***
**Sr**											1	0.620***
**Yield**												1

*** Significant at 0.05, 0.01 and 0.001 levels, respectively (n = 23).

Waterlogging caused an obvious decrease in yield (Table S1). The yield of each of 12 varieties tested under waterlogging was less than 20% of that under the control, while only 3 varieties under waterlogging had more than 75% of the yield in control. The yields of the majority of varieties (18/25) were lower by fifty percent of that of control plants.

Our results (Table S1) showed that compared with normal conditions all of the traits evaluated showed a decrease in response to waterlogging. Waterlogging resulted in plant mortality in most of the varieties. Four varieties (9.3%) had less than 20% of plants compared to the control, and 12 varieties (48%) had fewer than half plants of the control after waterlogging. Only 6 varieties (24%) had all of the plants alive under waterlogged conditions. All other traits were also decreased in response to waterlogging. Plant heights of 19 varieties (76%) were decreased more than 50% after waterlogging. Twenty varieties (80%) had only fewer than half of the branch height, primary branches, total branches and siliques of the control. Sixteen varieties (64%) had fewer than half of the siliques on the main shoot of the control. There were fewer than half the siliques on branches for 22 varieties (88%) after waterlogging. Sixteen varieties (64%) had only half of the seeds in a silique of the control. Waterlogging also caused inhibition of root growth. The length of the roots for 17 varieties (68%) after waterlogging was less than half of the control. The main shoot length of 21 (84%) varieties was less than half of that of the control.

A wide range of variation was observed for all the traits among the different varieties under waterlogging. The WTC of yield ranged from 6.8% in 9981 to 91.3% in ZS9 (Table S1). The variety “B108” had only 62 plants left after waterlogging, accounting for 13.8% of the control, while there were six varieties which had 450 plants as many as the control. WTC of *ph*, *bh* and *msl* ranged from 8.4% to 79.8%, 8.2% to 88.3% and 8.9 to 79.3%, respectively. WTC of *pbr* and *tbr* ranged from 9.1% to 85.9%, and 7.9% to 86.1%, respectively. WTC of *sqms*, *sqb*, and *sqp* varied from 8.9% to 94.4%, 5.2% to 74.8%, and 6.6% to 82.8%. 9981 had only 9.8% of root length of the control while HYZ9 had 92.7%.

### The effect on seedling establishment by waterlogging

To compare waterlogging tolerance of the 25 rapeseed varieties at the seedling establishment stage, the germinated seeds were subjected to normal conditions and hypoxia conditions. The root length, shoot length, and full weight were examined to evaluate waterlogging tolerance.

Under control conditions, there was a significant positive correlation between root length and full length as well as between shoot length and full weight. After waterlogging treatment was applied, there was a significant positive correlation between root length and shoot length, root length and full length, and shoot length and full length (Table 4).

**Table 4:** Correlation coefficients (*r*) between pairs of traits of seedlings at seedling establishment stage under control and waterlogging.

Control					Treatment				Waterlogging Tolerance Index			
	RL	SL	FL	FW	RL	SL	FL	FW	RL	SL	FL	FW
**RL**	1	−0.15	0.91***	−0.05	1	0.50**	0.96***	0.35	1	0.38	0.99***	0.78**
**SL**		1	0.27	0.40*		1	0.72***	0.23		1	0.45*	0.51**
**FL**			1	0.12			1	0.35			1	0.78***
**FW**				1				1				1

*, **, and *** Significant at 0.05, 0.01 and 0.001 levels, respectively (n = 23).

Besides, waterlogging decreased all of the four traits (Table S2). The average root length under waterlogging was only 33.8% of that of the control, while the average of shoot length, full length, and full weight under waterlogging was 67.9%, 40.7% and 71.2% of that under control, respectively. However, the changes in these parameters varied with varieties. ZS9 had the highest WTC of root length (61.6%), while that of Y208 was only 4.3%, and 76% of varieties (19/25) had less than half of root length under waterlogging when compared to that under the control. WTC of shoot length ranged from 40.0% to 99.9%. The largest full length rate was 65.9%, and the smallest one was only 17.9%. ZY821 represented the largest WTC of full weight, 93.5%, while that of Y208 was decreased to 44.5% by waterlogging.

### The effect on the seedlings by waterlogging

To compare waterlogging tolerance among the 25 rapeseed varieties at the seedling stage, the seedlings were waterlogged, and root length, shoot length, root weight, and shoot weight of the seedlings were evaluated after recovery.

Under normal conditions, correlation analysis among the traits indicated that there was significant positive correlation between root length and full length, and shoot length and full length (Table 5). A similar correlation was observed for root weight and full weight, and shoot weight and full weight. A significant positive correlation was also found for shoot length and full weight. After waterlogging, our analysis revealed that the full length was significantly related with root length, shoot length and root weight, while full weight was not significantly related with any of the other traits (Table 6 and 7).

**Table 5:** Correlation coefficients (*r*) between pairs of traits of seedlings at seedling stage under control.

	RL	SL	FL	RW	SW	FL
**RL**	1	0.04	0.84***	0.55***	−0.18	−0.03
**SL**		1	0.57***	0.18	0.61***	0.58***
**FL**			1	0.55***	0.18	0.29
**RW**				1	0.36	0.55***
**SW**					1	0.98***
**FL**						1

*** Significant at 0.05, 0.01 and 0.001 levels, respectively (n = 23).

**Table 6:** Correlation coefficients (*r*) between pairs of traits of seedlings at seedling stage under waterlogging.

	RL	SL	FL	RW	SW	FL
**RL**	1	−0.05	0.73***	0.54***	−0.04	0.09
**SL**		1	0.65***	0.27	0.62**	0.59**
**FL**			1	0.59**	0.39	0.47*
**RW**				1	0.53**	0.69***
**SW**					1	0.98***
**FL**						1

*, **, and *** Significant at 0.05, 0.01 and 0.001 levels, respectively (n = 23).

**Table 7:** Correlation coefficients (*r*) between WTC of pairs of traits of seedlings at seedling stage after waterlogging.

	RL	SL	FL	RW	SW	FL
**RL**	1	0.34	0.95***	0.29	0.01	0.15
**SL**		1	0.60**	−0.18	−0.26	−0.22
**FL**			1	0.18	−0.07	0.07
**RW**				1	0.66***	0.88***
**SW**					1	0.93***
**FL**						1

**, and *** Significant at 0.01 and 0.001 levels, respectively (n = 23).

Waterlogging inhibited the growth of seedlings to some extent (Table S3). Except for WTC of root weight, the average WTC of other traits ranged from 80% to 90%. The average WTC of root weight for all of the 25 varieties was 72.7%. WTC of root length, shoot length, and full length ranged from 66.0%∼99.9%, 68.4%∼99.6%, and 69.8%∼98.4%, respectively. WTC of root weight, shoot weight, and full weight ranged from 43.8%∼96.6%, 70.0%∼99.5%, and 65.1%∼98.9%, respectively.

### Relationship between yield and other traits under waterlogging

Possible correlations were analyzed after waterlogging stress between WTC of yield and other traits. Significant correlations were found between WTC of yield and other 17 evaluated traits, including root length (P<0.001), full weight (P<0.05), full length (P<0.01) at seed germination stage, shoot length (P<0.05), root length (P<0.01) and full length (P<0.01) at seedling stage, and all the evaluated traits when maturity in fields. By contrast, the WTC of shoot length at the seedling establishment stage was not significantly related to the WTC of yield in the field, while there were no significant correlations between WTC of those weight related traits at the seedling stage and WTC of yield in the field.

### Waterlogging tolerance evaluation of 25 rapeseed varieties

The ranking of waterlogging tolerance for the 25 genotypes was done on the basis of their performance in WTC of those traits at the seedling establishment and seedling stages, which were significantly correlated with the WTC of yield. The genotypes were ranked as 1–25 with ‘1’ being the most tolerant and ‘25’ being as susceptible based upon their abilities to withstand waterlogging conditions. The ranks were pooled to calculate an overall ranking of the genotypes on the basis of these traits (Table S4).

Screening at the seedling establishment stage identified four waterlogging-tolerant varieties, ZS9, ZY821, HYZ9, and YG2009, of which ZS9, YG2009, and HYZ9 were also identified as high-tolerant varieties in the field. HYZ62, H0102, HYZ6, HYZ9 and YG 2009 were identified to be waterlogging-tolerant at the seedling stage, and YG2009 was identified as the tolerant one in the field. Although some of the varieties, which were evaluated to be tolerant at the seedling establishment stage and seedling stage, were not in the top list of tolerant ones on yield, most of these varieties were above the media tolerance of all the varieties.

## Discussion

### There was significant difference of yield loss in rapeseed under waterlogging stress

The first step of breeding waterlogging tolerant rapeseed is to identify difference of tolerance in species. As a crop, yield is the most important trait for breeding. Waterlogging tolerance for rapeseed means less yield loss under waterlogging. In this study, we screened 25 varieties of rapeseed in fields for waterlogging tolerance.

As expected, waterlogging decreased the yield in all of the varieties of rapeseed. As shown in Table S1, most of these varieties were very sensitive to waterlogging, and yield loss reached more than fifty percent. According to the breeding and research history, problem of waterlogging did not attract a lot of attention before, and breeders have not focused on waterlogging tolerance in breeding. Moreover, there is little research on waterlogging stress in rapeseed. Breeding experiments are generally carried out under normal condition. Although the yield is excellent under normal condition, it declines significantly when the plants are subjected to waterlogging.

Although waterlogging caused yield loss in all of these 25 varieties, considerable natural variation was observed among rapeseed varieties. There was a significant decrease in yield of non-tolerant genotypes. However, the tolerant genotypes had relatively less reduction in the yield (Table S1). The most tolerant one, YG2009, showed only a 10% reduction in yield compared to that of the control. Our results suggested that substantial genetic variations existed in waterlogging tolerance in rapeseed. This difference opens up the possibility to study the underlying mechanisms and genes responsible for these variations.

In our study, waterlogging treatment not only caused yield loss in these varieties, but also led to the death of plants for most of these varieties as shown in Table S1. In a previous study in Arabidopsis, flooding over all the growth stage did not lead to the death of any plants [11]. In another study in Arabidopsis, media lethal times were estimated as a measure of tolerance only when Arabidopsis accessions were subjected to complete submergence [10]. In our study, the seedlings were recovered after being waterlogged for 14 days. This system led to considerable lethal rate for these varieties, revealing that rapeseed is very sensitive to waterlogging and seedling stage is a sensitive stage during plant ontogeny. After the plants encountered waterlogging at the seedling stage, these rapeseed varieties also showed a large diversity in survival ability. This is one reason different varieties had different yield loss after the stress, and it is also revealed by the results that the survival rate was significantly correlated with the yield index.

**Figure 1:**
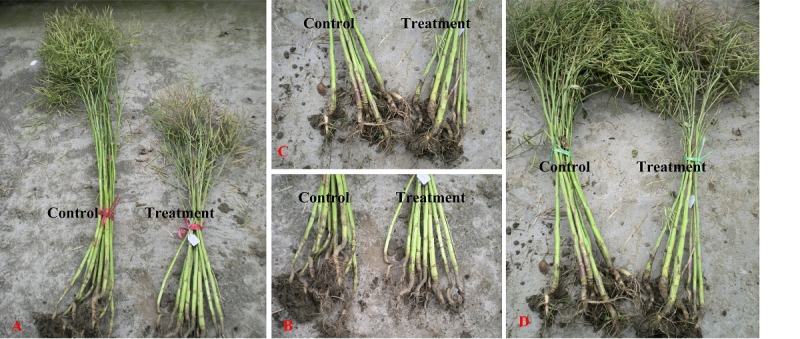
The effect of waterlogging on morphological traits of GH01 and ZS9 in field conditions. A: GH01 control and treated plants; B: GH01 control and treated roots; C: ZS9 control and treated roots; D: ZS9 control and treated plants.

Besides the differences in yield loss and survival rates among the 25 varieties, other morphological traits also decreased. Generally, the growth of these varieties were all inhibited after waterlogging in our study. This was also found in Arabidopsis, maize, soybean, *etc* and plants showed an inhibition of growth under waterlogging or submergence [10], [11], [19]. Taking plant height as an example in this study, 19 out of 25 varieties after waterlogging had only half of the plant height compared to that of control (Table S1), suggesting that waterlogging in rapeseed inhibits growth instead of promotion of growth which is revealed in some species, such as *Rumex palustris* [20] or deepwater rice [21]. The inhibition also displayed on other morphological traits (Figure 1). All of the varieties showed a decrease in root length when exposed to waterlogging stress. The aerial structures were also affected by the stress. Under waterlogging, the plant height was decreased, leading to a lower position of branch height, fewer branches, and shorter main shoot length. Certainly, the smaller plants after the stress had fewer siliques on the main shoot and siliques on a plant. Besides, seed number in siliques (*Ssq*) was also decreased as a result of waterlogging. According to these factors above, yield under waterlogging was decreased compared to that of the control. It seems the most important problem is decreased oxygen concentration in the soil in the case of excess water. This primarily affects the root system and, as a consequence, the above-ground growth. Under flash waterlogging, the plant restricts its growth and growth is still inhibited compared to the control even after removal of water. The stress eventually decreases yield for rapeseed. Similar to yield, the morphological traits of roots and aerial structure varied among the 25 varieties of rapeseed. Our results revealed that WTC of these traits were all significantly correlated with that of yield, indicating the tolerant one would have a strong ability to keep growth potential under waterlogging and would recover better from the stress (Figure 1). Our data further suggests a consistency between the selected morphological traits and yield in waterlogging tolerance of these genotypes until maturity. Waterlogging-tolerant plants could be evaluated by any of these morphological traits at maturity.

### Roots are the most sensitive part to waterlogging at the seedling establishment stage

Seedling establishment of rapeseed seeds in paddy fields that are either waterlogged or submerged is one instance in which rapeseed is exposed to hypoxia or even anoxia. Waterlogging which can impose stress on seedlings and has been shown to reduce crop yield [22], [23]. However, waterlogging poses a significant problem to seeds prior to emergence. We exposed the 25 varieties of rapeseed to waterlogging during the seedling establishment stage and evaluated the morphological traits after the recovery. Seedling establishment stage is a sensitive stage to waterlogging. Seeds of a vast majority of higher plant species fail to germinate under waterlogging stress and the germination rates decline dramatically, including soybean, pea (*Pisum sativum*), wheat, maize, and rice [24], [25]. Fatty seeds are more sensitive to hypoxia than starchy seeds because starchy ones can maintain a high energy metabolism even under the stress [26]. Rapeseed bears fatty seeds, therefore, waterlogging can cause low germination rates or even failure to germinate in several varieties. Although, the stress lasted for only 12 h in our study, it still caused extensive damage to the rate of germination.

The results also revealed that the length and weight of rapeseed seedlings decreased significantly under waterlogging during the seedling establishment stage. In some varieties of rice, the final length of coleoptiles can exceed the length of aerobic coleoptiles when germinated without oxygen [27]. Under the stress, rice seedlings elongate the coleoptile in an attempt to breach the surface of the water and thus allow oxygen transport to underwater tissues. On the other hand, in other varieties of rice and other species, waterlogging inhibits the growth of seedlings at the seedling establishment stage [28], [29]. Our study in rapeseed corroborated these results as the growth of rapeseed seedlings was inhibited under waterlogging. Besides, the tolerant varieties mean strong ability of seed germination and seedling growth under waterlogging and the inhibition was shown to a much lower extent in tolerant ones.

**Figure 2:**
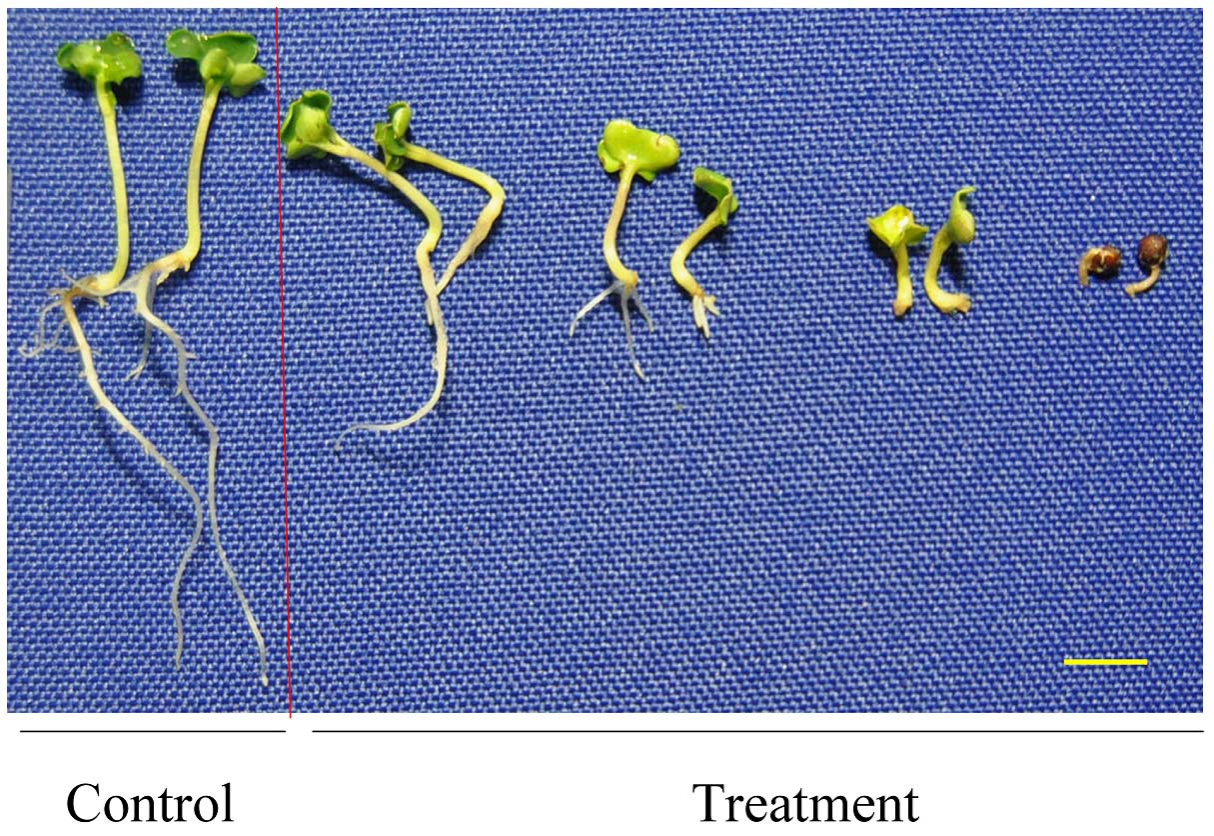
The effect of waterlogging on seedlings of 9981 at the seedling establishment stage. Yellow bars = 1.0 cm.

Although the seedlings of some varieties had shoots and leaves like a normal plant, they did not have radicles as a result of waterlogging (Figure 2). Instead, some adventitious roots were generated, which is an adaptive mechanism under waterlogging [30], [31]. In this seedling establishment stage, the root tips are very sensitive to hypoxia. The quick cell division and expansion is indeed oxygen requiring in the meristem tissues [32]. Meristems are the first target of stress and waterlogging stimulates root tip death.

### Waterlogging affected the length of seedlings at the seedling stage

Most of waterlogging-tolerance screening experiments were carried out at the seedling stage. In our study, the results showed that waterlogging led to the inhibition of growth for both roots and shoots for rapeseed seedlings (Table S3). Under waterlogging, most plants, such as Arabidopsis [10], [11], [33], barley [34], maize [14], soybean [35], [36], *etc*, produced smaller roots because of the anoxic conditions of the soil . This inhibition of growth of seedlings was because of a passive response to severe stress rather than an adaptive response, although Sub1A controls submergence tolerance through inhibition of growth under the stress in rice [37]. Waterlogging tolerance could be evaluated using the waterlogging tolerance coefficient (WTC = waterlogging treatment of each trait/control of each trait). In maize, Qiu performed QTL mapping associated with waterlogging tolerance using WTC during the seedling stage [38].

Similar to that in the fields or at seedling establishment stage, variations of tolerance were found for the six morphological traits at the seedling stage. Although the seedlings were waterlogged for 5 days, the extent of inhibition of growth at seedling stage was not as pronounced as that at the seedling establishment stage. The results revealed that the WTC of the root weight had the largest range of variation among all varieties. There can be attributed to two reasons: 1. WTC of root length varied among the 25 varieties under waterlogging; 2. adventitious roots were generated under waterlogging stress. Although the number of adventitious roots was not evaluated in our study, there should be differences in different varieties, leading to the difference in root weight.

### WTC of six traits at the other two development stages were significantly related to the WTC of yield

There are several kinds of systems for screening plants for waterlogging tolerance. Most of the previous studies dealt with tolerance at a specific growth-stage. In our study, three systems of screening for rapeseed over the ontogeny were performed. The growth of rapeseed was inhibited by waterlogging irrespective of the developmental stage. Moreover, the inhibition of growth led to the loss of yield in fields. Based on this idea, correlation between WTC of the yield and traits from two other development stages were analyzed. The results revealed that the WTC of six traits at the other two development stages were significantly related with the WTC of yield.

At the seedling establishment stage, the roots were very sensitive to waterlogging as described above. As expected, there was significant correlation between WTC of root length at the seedling establishment stage and yield in the field. The length of shoots was decreased by waterlogging, and the extent of decrease varied in different varieties (Table S2). However, the growth of shoots was not as sensitive as that of roots at the seedling establishment stage, not leading to correlation between WTC of this trait and yield. Radicle protrusion is the first stage in the emergence of rapeseed seedlings and the roots are generally much longer than shoots at this stage. Although the variation of shoot length was not correlated with yield, the full length and full weight of seedlings was significantly correlated with yield in fields for the roots were very sensitive to waterlogging stress.

**Figure 3:**
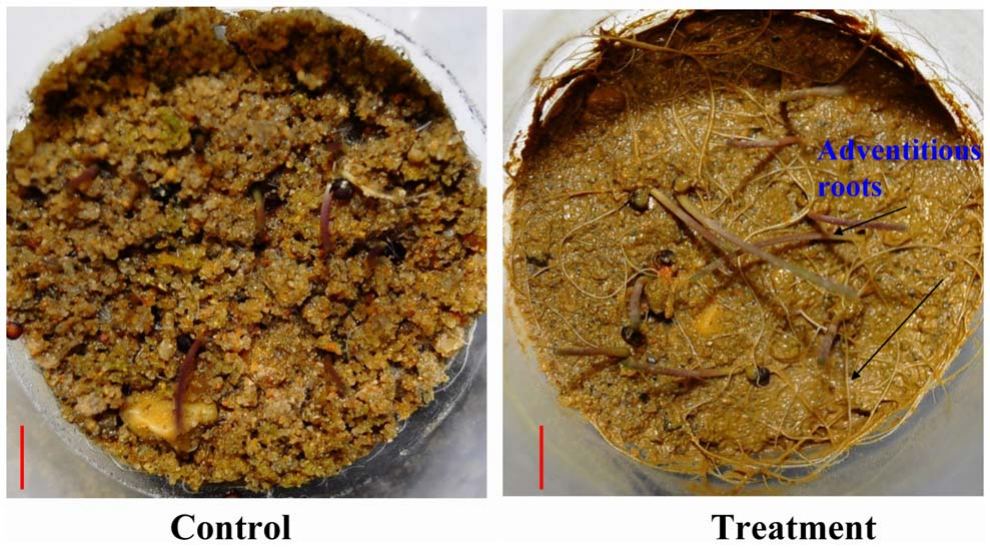
The adventitious roots of ZS12 induced by waterlogging at seedling stage. Red bars = 1.0 cm.

**Figure 4:**
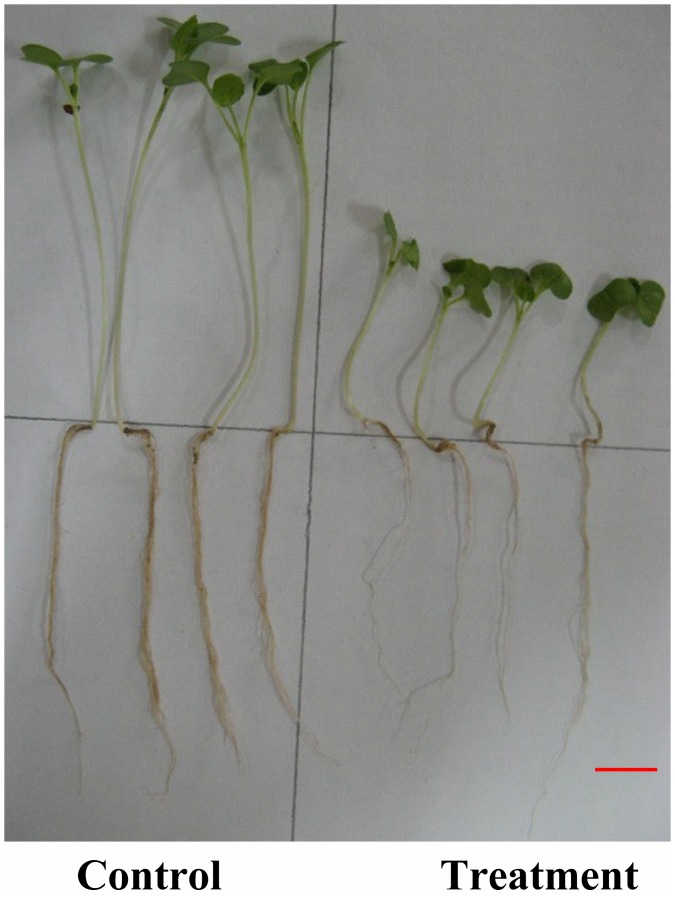
The effect of waterlogging on seedlings of B108 at the seedling stage.

At the seedling stage, it is interesting to note that the WTC of all the three traits of length were significantly correlated with the WTC of yield, while the WTC of the other three traits related with weight were not correlated with WTC of yield. Although the growth of main roots was inhibited by waterlogging, which led to the inhibition of shoots, the adventitious roots were induced by waterlogging stress (Figure 3). Despite of the decrease in weight of main roots under waterlogging, the WTC of root weight did not change linearly according to the tolerance ability of varieties. Besides, the WTC of shoot weight was also not significantly correlated with yield. Seedlings had several leaves at this stage (Figure 4), and the leaf growth was not decreased by waterlogging as the same as the length of shoots, leading to no significant correlation between WTC of shoot weight and yield.

### The advantage and disadvantage of the three systems for screening waterlogging tolerance

The three systems tested had their both advantages and disadvantages. Although yield is the most important trait from an agronomical point of view, the examination of yield and yield related traits under waterlogging stress is the most time-consuming step in the selection and testing process. Besides, it is difficult to reproduce the field conditions in every experiment, including the soil, stress extent, and the weather, etc. The major problem in waterlogging-related studies is that the observed tolerance is specific to a given environment. Reproducible experiments are possible only if the genotypes are tested under controlled water and weather conditions. Some studies reporting the effect of waterlogging on yield traits were performed under controlled climatic conditions [39]. In such situations, all of the growth conditions can be controlled. However, these are cost intensive and the capacity of the experiments is limited. To guarantee the selection of suitable genetic sources for breeding and/or to clarify the role of stress-induced genes in the regulatory pathways, more accurate and convenient testing systems need to be elaborated.

Seedling test conducted in controlled conditions is the most used method to screen waterlogging tolerance and provide a lot of data about physiological or molecular aspects [17], [40]. Our results suggest that some traits due to waterlogging stress were induced at early growth stages and could predict yield loss in the fields. Although the labor of growing seedlings in containers in greenhouse is much less compared to that in the field, it is still very costly and laborious. The biggest problem is that the variation of seedling traits under waterlogging compared to waterlogging and the difference among different varieties was not very large, despite the seedlings were waterlogged for 5 days.

It is fortunate to find WTC of some traits at seed germination stage significantly related with WTC of yield in our study. Germination tests are efficient for selecting lines from large numbers of accessions in different species. They require little investment and provide a large number of data within several days. This simple and rapid method can be used for mass testing.

If plants have a consistent pattern of waterlogging tolerance at all growth stages, the tolerant ones could be selected at any stage. Based on the ranking analysis (Table S4), although some varieties with median tolerance were found to be not consistently superior across the three growth stages, those varieties of extreme tolerance or sensitivity showed consistency in their tolerance during the ontogeny of the whole plant, suggesting that the screening at the early stage would provide a suitable basis for the initial selection of a large number of germplasm entries or breeding populations for waterlogging tolerance.

## Conclusion

In summary, the variations in waterlogging tolerance with respect to yield were found among different varieties. These variations provide an important basis for the further development of breeding materials that are more tolerant to waterlogging stress. According to the fact that flash waterlogging restricts plant growth and growth is restored after removal of the stress.

The assessments of genotypes for physiological parameters at the three growth stages suggest that there were differences in waterlogging tolerance at all the development stages. Correlation analysis revealed that there was consistency in the waterlogging tolerance of the selected genotypes until maturity, and good waterlogging tolerance at the germination stage and seedling stage can guarantee tolerance in later stages. The waterlogging-tolerant plants could be selected at any stage, and the selections would be more effective at the seed germination stage. Our study provides a method for screening plants for waterlogging tolerance, which helps in the initial selection of a large number of germplasms or breeding populations for waterlogging tolerance and determine their potential utility in crop-improvement.

## Supporting Information

Table S1 The effect of waterlogging on the 25 varieties in fields.
(XLS)

Table S2 The effect of waterlogging on the 25 varieties at seedling establishment stage.
(XLS)

Table S3 The effect of waterlogging on the 25 varieties at seedling stage.
(XLS)

Table S4 Performance-based ranking of the 25 varieties for yield significantly-related traits at seedling establishment stage and seedling stage.
(XLS)
